# Autosomal dominant polycystic kidney disease in Colombia

**DOI:** 10.1186/s12882-023-03266-3

**Published:** 2023-07-17

**Authors:** Jessica T. Camargo, Camilo A González, Lina Herrera, Nancy Yomayusa-González, Milciades Ibañez, Ana M. Valbuena-García, Lizbeth Acuña-Merchán

**Affiliations:** 1 Fundación Universitaria Sanitas, Bogotá, D.C Colombia; 2 Unidad Renal, Clínica Colsanitas, Calle 127 No 20-78 Piso 2, Bogotá, D.C Colombia; 3 Cuenta de Alto Costo, Fondo Colombiano de Enfermedades de Alto Costo, Bogotá, D.C Colombia; 4 Global Institute of Clinical Excellence, Keralty, Bogotá, D.C Colombia; 5 Instituto de Investigación, Fundación Universitaria Sanitas, Bogotá, DC Colombia

**Keywords:** Chronic kidney failure, Polycystic kidney disease, Renal replacement therapy, Dialysis, Kidney transplant

## Abstract

**Background:**

Autosomal dominant polycystic kidney disease (ADPKD) is the most common genetic cause of chronic kidney disease (CKD) that requires dialysis. Knowing geographical clusters can be critical for early diagnosis, progression control, and genetic counseling. The objective was to establish the prevalence, geographic location, and ethnic groups of patients with ADPKD who underwent dialysis or kidney transplant in Colombia between 2015 and 2019.

**Methods:**

We did a cross-sectional study with data from the National Registry of Chronic Kidney Disease (NRCKD) managed by the High-Cost Diseases Fund (*Cuenta de Alto Costo* [CAC] in Spanish) between July 1, 2015, and June 30, 2019. We included Colombian population with CKD with or without renal replacement therapy (RRT) due to ADPKD. Crude and adjusted prevalence rates were estimated by state and city.

**Results:**

3,339 patients with ADPKD were included, period prevalence was 9.81 per 100,000 population; there were 4.35 cases of RRT per 100,000 population, mean age of 52.58 years (± 13.21), and 52.78% women. Seventy-six patients were Afro-Colombians, six were indigenous, and one Roma people. A total of 46.07% began scheduled dialysis. The highest adjusted prevalence rate was in *Valle del Cauca* (6.55 cases per 100,000 population), followed by *Risaralda*, and *La Guajira.* Regarding cities, *Cali* had the highest prevalence rate (9.38 cases per 100,000 population), followed by *Pasto*, *Medellin*, and *Bucaramanga*.

**Conclusions:**

ADPKD prevalence is lower compared to Europe and US; some states with higher prevalence could be objective to genetic prevalence study.

## Background

Autosomal dominant polycystic kidney disease (ADPKD) is characterized by the gradual growth of fluid-filled cysts in both kidneys, leading to increased size, loss of function, and eventually kidney failure [1]. It is the first hereditary cause and the fourth most common cause of the need for renal replacement therapy (RRT) worldwide [2–5]. ADPKD has been described in all ethnic groups, with variable regional prevalence [6–9]. Mutations in the PKD1 and PKD2 genes are the most frequent etiology of the disease [2]. PKD1 is more common and causes up to 85% of cases. However, spontaneous mutations may occur in up to 5% of cases [1, 10]. Kidney failure leads to up to 70% of patients requiring RRT between 40 and 70 years of age, with earlier ages at onset in cases with PKD1 mutations compared to those with PKD2 mutations [4], generating high costs for the health system and reducing the health-related quality of life [2, 4].

Estimating the prevalence of ADPKD is difficult owing to a long asymptomatic period and underdiagnoses caused by the lack of an adequate approach to detecting the disease. The genetic prevalence in small cohorts is as high as 1 per 1,000 population, but population-based studies show a lower prevalence [11]. Latin America does not have enough information in this field. Colombia, where we have multiple origins and genetic mixtures, probably shows a different prevalence than the rest of the world [12].

The main objective of this study was to determine the prevalence of ADPKD in Colombia. However, to avoid selection bias due to the asymptomatic period and underdiagnoses, we included individuals with ADPKD receiving RRT to compare the geographical prevalence and describe their clinical characteristics between 2015 and 2019.

## Methods

We conducted a cross-sectional observational study with data from the National Registry of Chronic Kidney Disease (NRCKD) managed by the High-Cost Diseases Fund (*Cuenta de Alto Costo* [CAC] in Spanish) from July 1, 2015, to June 30, 2019. NRCKD is an official registry with a national scope within the framework of the general system of social health insurance (SGSSS in Spanish), taking into account that approximately 97% of the population is covered by this system [13]. All grades (1 to 5 by Kidney Disease, Improving Global Outcomes KDIGO) of CKD must be reported to the NRCKD in Colombia. CKD was defined as glomerular filtration rate (eGFR) less than 60 mL/min/1.73 m2 for at least three months, albumin-to-creatinine ratio > 30 mg/g in two of three spot urine specimens, or abnormal kidney structure by images [14]. The reporting process is legally mandatory [15]. The inclusion criteria were residents of Colombia who were reported to the NRCKD with a diagnosis of CKD with or without RRT (dialysis or kidney transplant) and ADPKD. However, the descriptive analysis was performed only for patients on RRT, contemplating the limitation expected by the underreporting of patients without RRT.

Each case was identified with a unique registration number to protect the personal information of the participants. Additionally, a structured audit process was carried out to ensure the quality of the information provided by comparing it with the medical records.

The study variables were divided into demographic (age, sex, type of health insurance, ethnicity, states and cities of residence, and follow-up time) and clinical variables (weight, height, serum creatinine, estimated glomerular filtration rate (eGFR) at diagnosis, time since diagnosis of CKD, time since diagnosis of CKD stage 5, time since the start of RRT and its initial modality, potential kidney transplant evaluation and vital status). To ensure the validity and reliability of this study, all patients who met the inclusion criteria were included.

### Statistical analysis

The qualitative data is informed as proportions, and the quantitative as the means, medians, and standard deviation or interquartile ranges (IQR). Crude prevalence is presented. The results were presented by georeferenced departments and municipalities. The prevalence was studied for four consecutive years (2015–2019); for each period, cases were included from July 1st of the previous year to June 30th of the year under study. The period prevalence was estimated by taking all new and pre-existing cases of ADPKD in the four years, over the mean or mid-interval population. Data analysis was performed with the statistical package STATA® 13.0 (StataCorp®) and georeferenced with the software Qgisnciv ® 3.2. The prevalence was measured with Microsoft® Excel® version 2019.

## Results

A total of 3,339 patients with ADPKD were reported NRCKD (overall period prevalence of 9.81 cases per 100,000 population), of which 1,476 patients were on RRT, for a period crude prevalence between 2015 and 2019 of 4.35 cases per 100,000 population (Table 1).

**Table 1:** Crude prevalence of ADPKD per 100,000 population

	2016	2017	2018	2019	2015–2019
Adults with ADPKD	7.26	9.14	9.12	9.31	9.81
Adults with ADPKD on RRT	3.53	3.98	3.84	3.92	4.35

For the patients on RRT, the mean age at the time of this study was 55.43 years (± 12.78 years), and the mean age at the start of RRT was 52.58 years (± 13.21 years). The female was predominant, with 779 patients (52.78%). (Table 2). Regarding ethnicity, among the patients with ADPKD, there were 76 Afro-Colombians patients (5.15%), six indigenous patients (0.41%), and one Roma patient (0.07%), and the remaining 1,393 patients (94.38%) did not have a specific ethnic assignment. Based on the 2018 Colombian ethnicity census, the prevalence of ADPKD on RRT was 2.1 cases per 100,000 population for Afro-Colombians, 0.31 cases per 100,000 population for indigenous, and in the 2,649 (0.008% of the Colombian population) auto-nominated Roma people or gypsy, an overestimated prevalence of 37.7 cases per 100,000 population.

Upon initiation of RRT, only 15.58% belonged to a CKD program. However, 680 patients (46.07%) had a scheduled admission to their first RRT visit, 458 (31.03%) started dialysis by emergency service, and the rest were not reported. The number of deaths during the study period was 226 (15.31%).

At the end of the study, on June 2019, 725 patients were on hemodialysis, and 271 were on peritoneal dialysis. That is, around 29 of every 1,000 patients on dialysis had ADPKD. Functioning kidney transplants were reported in 480 patients (32.52% of RRT patients).

**Table 2:** Demographic and clinical characteristics of patients with ADPKD on RRT, Colombia 2016–2019

Description	N = 1,476
**Demographics**	
Age at study onset in years, mean (SD)	55.43 (12.78)
Age at onset of RRT in years, mean (SD)	52.58 (13.21)
Female sex, n (%)	779 (52.78)
**Healthcare insurance affiliation**	
Contributory regime, n (%)	1,032 (69.9)
Subsidised regime, n (%)	409 (27.71)
Exceptions (military, police, etc.), n (%)	31 (2.1)
Special (*Ecopetrol*), n (%)	3 (0.2)
**Ethnicity**	
Indigenous, n (%)	6 (0.41)
Black, mulatto, or Afro-Colombian people, n (%)	76 (5.15)
**Clinical characteristics**	
Weight in kg, mean (SD)	65.39 (13.79)
Size in cm, mean (SD)	163.02 (9.35)
BMI in kg/m2, mean (SD)	24.51 (4.27)
Serum creatinine level at program admission in mg/dl, median (IQR)	5.29 (1.5-9)
Time with stage 5 CKD in years, median (IQR)	5.46 (2.66–9.36)
Dialysis vintage in years, median (IQR)	4.32 (2.53-7)
Kidney transplant waitlist, n (%)	735 (49.8)
Contraindication for kidney transplantation, n (%)	262 (17.75)
No data about the kidney transplant waitlist, n (%)	479 (32.45)
**Renal replacement therapy**	
Hemodialysis, n (%)	725 (49.12)
Peritoneal dialysis, n (%)	271 (18.36)
Functioning kidney transplant, n (%)	480 (32.52)
Deaths from any cause, n (%)	226 (15.31)

**Fig. 1:**
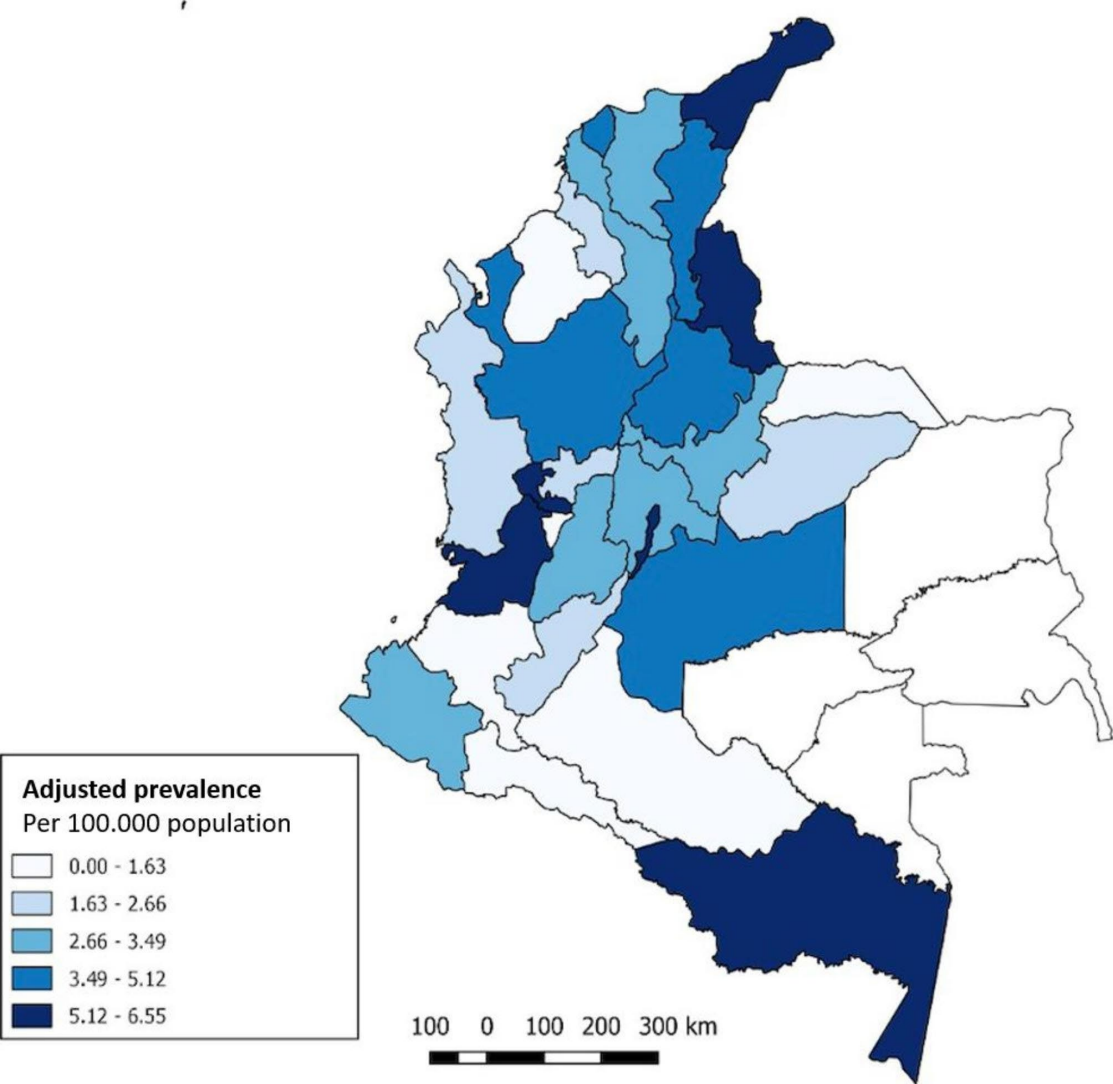
ADPKD period prevalence on RRT by states (*Departamentos* in Spanish), 2015–2019. (Data source: NRCKD)

Regarding the geographic distribution of the disease by state (*Departamentos* in Spanish for Colombia) (Fig. 1), the highest standardized prevalence rates were found in *Valle del Cauca* (6.55 cases per 100,000 population), *Risaralda* (6.48 cases per 100,000 population) and *La Guajira* (5.56 cases per 100,000 population). The lowest prevalence rates were reported in Quindío and Caquetá, with 0.57 and 0.08 cases per 100,000 population, respectively. Table 3 shows the adjusted prevalence of CKD on RRT from any cause, caused by ADPKD, and the overall mortality from any cause at some states of extremes of ADPKD prevalence. La Guajira has the highest proportion of ADPKD patients on RRT (10.48%). Overall adjusted mortality of CKD patients on RRT was higher in Valle del Cauca.

**Table 3:** Adjusted prevalence of CKD on RRT from any cause, caused by ADPKD, and CKD overall mortality in 2019 (Per 100.000 population)

	CKD on RRT prevalence			CKD on RRT Mortality	
State	All causes		ADPKD, prevalence (%)	Death from any cause	
La Guajira		53.05	5.56 (10.48)		5.82
Risaralda		86.62	6.48 (7.48)		7.73
Valle del Cauca		111.07	6.55 (5.90)		11.37
Bogotá D.C		92.11	5.2 (5.65)		8.66
Quindio		92.61	0.57 (0.62)		8.99
Caqueta		48.92	0.08 (0.16)		6.69

Data source: NRCKD

Regarding the main cities, Cali had the highest prevalence rate (9.38 cases per 100,000 population), followed by Pasto (8.81 cases per 100,000 population) and Medellin (7.30 cases per 100,000 population).

## Discussion

CKD is a public health problem that affects more than 10% of the general population worldwide [16]. ADPKD is the leading hereditary cause [16], responsible for up to 10% of patients with kidney disease on RRT [17, 18]. In Colombia, in 2019, more than 925,996 patients with CKD were reported, with a possible sub-estimated prevalence of 1.8%, of which 4.9% had End-stage Kidney Disease (ESKD) [19]. We suppose the early diagnosis of CKD in Colombia is reduced because of the silent period of the disease, young population composition, cultural perception of preventive medical consultation, and, despite the almost universal health system coverage, dispersed areas have less access to quality health services [20, 21].

Consultations with physicians, including both generalists and specialists in Colombia for 2019 was 2.6 doctor consultations per person, lesser than the average 5.3 for OECD members [22]. Vulnerable socio-economic groups still have obstacles to accessing health care. However, preventive health care consultations in the poorest quintile are rising. Access to healthcare services is more difficult in rural areas inhabited by indigenous communities, who may have beliefs, traditions, and models of healthcare that are different from the Western healthcare model. Indicators such as maternal and neonatal mortality rates tend to be higher in rural areas and for specific population groups (e.g., indigenous and Roma people) [20–22].

Those explain our low ADPKD period prevalence of 9.81 cases per 100,000 population compared to the majority of high-income countries, such as in Europe, where the prevalence is 39.6 per 100,000 population [7], or in the US, which has a prevalence of 42.6 per 100,000 population [11]. On the other hand, in Colombia, the prevalence of CKD on RRT are increasing from 77 to 86.13 per 100,000 population between 2016 and 2019 [23], but is at least 2.5 times less than in the US for 2019 [24]. According to our estimation, ADPKD patients who undergo dialysis or kidney transplant was 4.35 cases per 100,000 population, lower than that reported by the ERA-EDTA Registry on RRT from 12 European countries between 1991 and 2010, it shows an increasing ADPKD prevalence from 5.68 to 9.11 per 100,000 population [4].

In Colombia, every 29 patients out of 1,000 on RRT have ADPKD. This prevalence is comparable to Alves EF et al. that showed a prevalence of 10 per 1,000 patients on hemodialysis [9]. Likewise, in Africa, the frequency in the University Clinic of Nephrology and Hemodialysis of Cotonou was estimated at 18 per 1,000 population [25], and in Taiwan, it was estimated at 19 per 1,000 population on dialysis [26]. Moreover, in 2020 in the US, 50 per 1,000 patients were on RRT, 56% were on hemodialysis, 27% were on peritoneal dialysis, and 17% had functional kidney transplantation [24].

The ADPKD patients in Colombia started RRT at an average age of 52.8 years, and these patients were more likely to be women, with around 50% on hemodialysis, 18% on peritoneal dialysis and 32% who had kidney transplantation, similar to the US for the same year. For the US, the median age at the start of RRT between 2001 and 2010 was 55.6 years without changes, but RRT was more frequent among males [3]. For Europe, the mean age at the start of RRT increased from 56.6 to 58.0 years (p < 0.01) between 1991 and 2010. The increased age at onset of RRT is most likely due to increased access for elderly ADPKD patients or lower competing risks prior to the start of RRT [4].

Our study showed that there is a wide epidemiological variability in the different geographical regions of our country, with a high prevalence in the states and main cities of the periphery, probably related to the effect of georeferenced family clusters, health care access, and the centralization of RRT. That last hypothesis explain the highest prevalence of CKD of any cause in *Valle del Cauca* as well as for ADPKD in TRR. This regional difference coincides with observations in other latitudes, where similar studies have been carried out [27, 28]. The lowest prevalence was found in Quindío and Caquetá, with 0.57 and 0.08 cases per 100,000 population, respectively. Interestingly, Quindío showed one of the lowest prevalence rates compared to Risaralda, which was the department with the second highest prevalence rate (6.48 cases per 100,000 population). These two states have minimal geographical separation, and their cultures are similar; migration during the colonial period likely explains such a markedly different distribution. A low prevalence of ADPKD among indigenous should be caused by a low health care consultation or less probably by fewer frequent genetic PKD mutations. These hypotheses can be clarified with a genetic study, which allows the description of the prevalence of mutations.

Some strengths of this study is the data based on a nationwide registry with audited medical records, estimated prevalence by states and cities; and in the future, it will allow monitoring and even genotyping to group patients and families according to the risk of disease progression, taking into account the mutational profile and establishing primary and secondary prevention measures and early therapies to prevent progression to dialysis or kidney transplantation [29].

### Limitations

Given the secondary source of data, a standardized diagnosis of the disease is lacking. For NRCKD the diagnosis was based on nephrologist clinical judgment. Additionally, we lack information on approximately 3% of the population, which is not affiliated to the health care system. Unfortunately NRCKD doesn’t have a detailed mortality rate and causes of CKD patients. Similarly, we do not follow up on relatives of reported patients, it is the responsibility of the health provider and insurer within the health system model of Colombia, to actively search for relatives affected by the disease, and to carry out the corresponding notification and intervention.

## Conclusions

The prevalence of patients with ADPKD on RRT is lower than in Europe and the US. However, similar to known to Africa and Latin America; the areas with the highest prevalence rates in Colombia are found in *Valle del Cauca, Risaralda* and *La Guajira*. It is imperative to carry out the genetic geographic characterization of ADPKD by region.

## Data Availability

The data that support the findings of this study are available from High Cost Diseases Fund (*Cuenta de Alto Costo* [CAC] in Spanish), but restrictions apply to the availability of these data, which were used under license for the current study, and so are not publicly available. Data are however available from on reasonable request via email to the coauthor: Ana M Valbuena-García: avalbuena@cuentadealtocosto.org.

## List of abbreviations

ADPKD: Autosomal dominant polycystic kidney disease
CKD: chronic kidney disease
NRCKD: national registry of chronic kidney disease
CAC: High Cost Diseases Fund (*Cuenta de Alto Costo* in Spanish)
RRT: renal replacement therapy
US: United States of America
eGFR: estimated glomerular filtration rate
IQR: interquartile range
CIOMS: Council of International Organizations of Medical Sciences
SD: Standard deviation
ERA-EDTA: European renal association - European Dialysis and Transplant Association Registry
ESKD: End-stage kidney disease
